# Evidence of asymptomatic submicroscopic malaria in low transmission areas in Belaga district, Kapit division, Sarawak, Malaysia

**DOI:** 10.1186/s12936-019-2786-y

**Published:** 2019-05-02

**Authors:** Adela Ida Jiram, Choo Huck Ooi, José Miguel Rubio, Shamilah Hisam, Govindarajoo Karnan, Nurnadiah Mohd Sukor, Mohd Mafie Artic, Nor Parina Ismail, Nor Wahida Alias

**Affiliations:** 1 Parasitology Unit, Infectious Diseases Research Centre, Institute for Medical Research, Ministry of Health Malaysia, Jalan Pahang, 50588 Kuala Lumpur, Malaysia; 2 Vector Borne Diseases Section, Sarawak Health Department, Ministry of Health Malaysia, Diplomatik Road, Off Bako Road, Petra Jaya, 93050 Kuching, Sarawak Malaysia; 3 Malaria & Emerging Parasitic Diseases Laboratory, Parasitology Department, National Centre of Microbiology, Instituto de Salud Carlos III (ISCIII), Carretera de Majadahonda - Pozuelo, km. 2,200, Majadahonda, 28220 Madrid, Spain

**Keywords:** Submicroscopic, Malaria, Asymptomatic, Belaga, Sarawak, Low transmission, *Plasmodium*

## Abstract

**Background:**

Malaysia has declared its aim to eliminate malaria with a goal of achieving zero local transmission by the year 2020. However, targeting the human reservoir of infection, including those with asymptomatic infection is required to achieve malaria elimination. Diagnosing asymptomatic malaria is not as straightforward due to the obvious lack of clinical manifestations and often subpatent level of parasites. Accurate diagnosis of malaria is important for providing realistic estimates of malaria burden and preventing misinformed interventions. Low levels of parasitaemia acts as silent reservoir of transmission thus remains infectious to susceptible mosquito vectors. Hence, the aim of this study is to investigate the prevalence of asymptomatic submicroscopic malaria (SMM) in the District of Belaga, Sarawak.

**Methods:**

In 2013, a total of 1744 dried blood spots (DBS) were obtained from residents of 8 longhouses who appeared healthy. Subsequently, 251 venous blood samples were collected from residents of 2 localities in 2014 based on the highest number of submicroscopic cases from prior findings. Thin and thick blood films were prepared from blood obtained from all participants in this study. Microscopic examination were carried out on all samples and a nested and nested multiplex PCR were performed on samples collected in 2013 and 2014 respectively.

**Results:**

No malaria parasites were detected in all the Giemsa-stained blood films. However, of the 1744 samples, 29 (1.7%) were positive for *Plasmodium vivax* by PCR. Additionally, of the 251 samples, the most prevalent mono-infection detected by PCR was *Plasmodium falciparum* 50 (20%), followed by *P. vivax* 39 (16%), *P. knowlesi* 9 (4%), and mixed infections 20 (8%).

**Conclusions:**

This research findings conclude evidence of *Plasmodium* by PCR, among samples previously undetectable by routine blood film microscopic examination, in local ethnic minority who are clinically healthy. SMM in Belaga district is attributed not only to *P. vivax*, but also to *P. falciparum* and *P. knowlesi.* In complementing efforts of programme managers, there is a need to increase surveillance for SMM nationwide to estimate the degree of SMM that warrant measures to block new transmission of malaria.

## Background

Malaysia declared its aim to eliminate malaria with a goal of achieving zero local transmission by the year 2020. However, malaria continues to be a public health burden especially in the interior parts of the country, which accounts for a considerable number of cases treated at health clinics and admissions to hospitals. Although both *Plasmodium vivax* and *Plasmodium falciparum* are prevalent, in recent years, *Plasmodium knowlesi* has been recognized as the infective agent for an increasing number of cases, especially in Malaysia. In 2017, the country reported a total of 508 cases (local and imported) of the human type of malaria, down substantially from 6141 cases in 2010 [1]. However, up to 88% (3614/4114) of the country’s malaria cases are attributable to *P. knowlesi*, while *P. vivax* and *P. falciparum* accounts for 7% and 4% of total cases, respectively. The remaining 0.7%, 0.4% and 0.1% accounts for *P. ovale*, *P. malariae* and mixed infections respectively [2]. Sixty-eight percent of total malaria cases are found in Malaysian Borneo, in the states of Sabah and Sarawak. The remaining one-third (32%) of cases occur in Peninsular Malaysia, in the central, south-eastern and northern coastal regions.

Despite the decline in the number of malaria cases in the country, there have been reports of vivax and falciparum malaria cases in low endemic areas especially in the rural settings in Sarawak [2]. Nonetheless, all the human malaria cases are imported. Sarawak recorded a decline in malaria reported cases from 2802 in 2010 to 1442 cases in 2017 with 212 cases of human malaria and 1225 cases of zoonotic malaria where Kapit division registered the most number of malaria cases with 565 (all *P. knowlesi*). Kapit Division consists of three districts (Kapit, Song and Belaga). Malaysia is home to many isolated indigenous tribal groups that do not generally have the same level of access to health care as the rest of the population. Many indigenous people use traditional remedies before seeking care in a health facility, which can delay treatment. Many of these groups live within the forest or forest-fringe areas, where the vector ecology and transmission patterns of malaria present a unique challenge for vector control management [3].

At all levels of *Plasmodium* transmission, there are individuals with submicroscopic infection present in the population. The relative proportion of submicroscopic and microscopic infections varies between settings, depending on factors such as age, transmission intensity and immunity. In low transmission settings, submicroscopic infections may represent a significant fraction of infections, but the major determinants of the contribution of submicroscopic infections to malaria transmission are not clear. Submicroscopic infections are prevalent both in “stable” low endemic areas and in those areas experiencing recent reductions in transmission. It has previously been reported that in low transmission settings, the proportion of asymptomatic individuals is less than that in areas of greater transmission severity. Nonetheless, even in low transmission areas the asymptomatic cases make up for 60% of the infected population.

Despite rarely causing clinical disease, submicroscopic malaria infections can contribute to malaria transmission. Experimental evidence has demonstrated that individuals with submicroscopic infections are capable of infecting mosquitoes; while these individuals may infect fewer mosquitoes than individuals with higher parasite counts, the high numbers of individuals with low-density infections may lead them to contribute substantially to malaria transmission [4]. Understanding the prevalence of these infections and the extent to which they contribute to malaria transmission is critical for designing effective malaria control programmes. Achieving malaria elimination requires targeting the human reservoir of infection, including those with asymptomatic infection. Smear-positive asymptomatic infections detectable by microscopy are an important reservoir because they often persist for months and harbour gametocytes, the parasite stage infectious to mosquitoes. However, many asymptomatic infections are submicroscopic and can only be detected by molecular methods. This was evident in a paper by Jiram et al. [5], which indicates that the use of molecular method is imperative to show evidence of asymptomatic submicroscopic malaria within the Orang Asli (aboriginal) community. In this study, all samples were negative by microscopic examination. The use of molecular biology for the diagnosis of malaria has proved to be highly sensitive for the detection of the parasite, but the equipment required is not widely available in many of the endemic areas and the protocol is more complex and requires highly skilled personnels. The use of the polymerase chain reaction (PCR), however, is extremely useful for decision making in disease control and treatment, for example, for the detection of mixed infections which play a modulatory role in the severity of the symptoms [6–8]. The greatest advantage, however, is that the ultra-sensitive PCR now has ability to detect infections with parasitaemia as low as 22 parasites/ml blood [9, 10]. As malaria elimination programmes pursue mass screening and treatment of asymptomatic individuals, further research should strive to define the degree to which submicroscopic malaria contributes to the infectious reservoir and, in turn, what diagnostic detection threshold is needed to effectively interrupt transmission. Asymptomatic malaria is prevalent in malaria endemic regions and has become a serious threat as efforts towards eliminating malaria transmission are increasing [11].

Data on submicroscopic parasitaemia is needed to better understand disease progression and its impact on targets set in the National Malaria Elimination Programme. Current evidence is insufficient for understanding the contribution of low-density SMM infections to onward transmission to human populations. Intervention trials to directly assess the effect of identifying and treating low-density infections are warranted. Presently, limited studies on asymptomatic submicroscopic malaria are carried out in Malaysia. Both parasite and host factors will influence whether infections remain asymptomatic or become symptomatic and potentially life threatening. In order for Malaysia to achieve the national goal of malaria elimination by 2020, it is important to understand that asymptomatic submicroscopic infection plays an important role in malaria transmission and that interventions to target this parasite reservoir may be needed in both low- and high-transmission areas. Currently, there is a lack of information and investigation carried out on the outcome of submicroscopic asymptomatic malaria in the district of Belaga and in this region. Hence, the objective of this study is to investigate the prevalence of submicroscopic malaria among asymptomatic individuals from a low transmission area in the District of Belaga, Sarawak.

## Methods

### Study area and sample collection

**Fig. 1:**
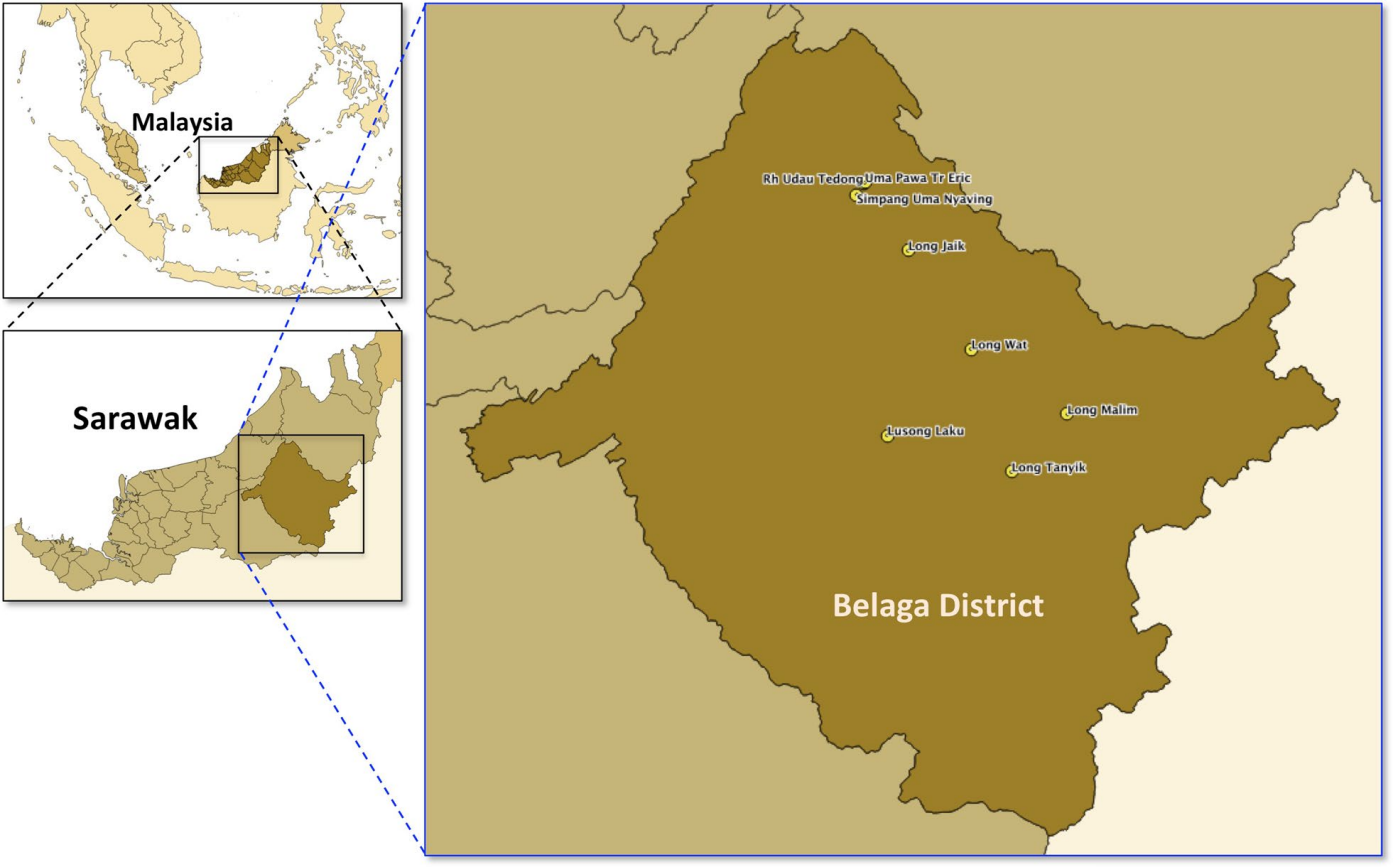
Map of Belaga District with the location of the 8 localities of longhouses in this study

In January 2013, the Vector Borne Disease Control Programme and Sarawak Department of Health has requested the Parasitology Unit of Institute for Medical Research (IMR) to assist in the detection of suspected SMM during a malaria outbreak investigation carried out in the district of Belaga, in Kapit, Sarawak. Belaga is located in the upper reaches of the Rajang River, some 120 km northeast of Kapit but considerably further on the river and slightly less than 100 km from the South China Sea coast near Bintulu. Belaga is considered a good place to start exploring the Sarawak’s interior. There are many ethnic minorities co-habituating riversides such as the Kenyah and Kayan longhouses along the Balui and Belaga rivers, and along the Rejang Rivers are the Punan, Sekapan, Kejaman and Tanjung longhouses. The total population in Belaga in 2013 was 39,400, with the malaria incidence rate of 2.9 per 1000 population. Subsequently, in 2014 and 2015, the malaria incidence rate was reduced to 2.7 and 1.6 per 1000 population, respectively. Despite continuous efforts to eradicate and eliminate malaria in this district, the infection persists. With the aim to demonstrate the presence of submicroscopic malaria in Belaga, a total number of 1744 dried blood spot samples (DBS) were collected during the routine Active Case Detection (ACD) from 8 localities within the district of Belaga in Sarawak, namely Long Malim, Long Wat, Long Jaik, Long Tanyit, Lusong Laku/SK Lusong Laku, Rh Udau Tedong, Uma Pawa Tr Eric, and Simpang Uma Nyaving (Fig. 1). DBS were sent to the Parasitology Unit in the IMR for molecular identification. Blood films were examined by microscopists from the Sarawak Department of Health. Subsequently, in 2014, further investigation was carried out in two localities (Long Malim and Long Wat) which were selected based on the presence of PCR-diagnosed *P. vivax* detected during the 2013 outbreak investigation activity. However, the residents from Long Malim and Long Wat were currently relocated to a new Tegulang Resettlement Site. The total population of Long Malim and Long Wat in 2014 was 253 and 321, with an incidence rate of 4.0 and 0 per 1000 population, respectively. In 2014, both longhouses reported zero malaria incidence by microscopic examination. A total of 112 and 139 blood samples in EDTA and blood films were collected from Long Malim and Long Wat, in October 2014 respectively.

### Blood sample collection and preparation

Finger prick blood (0.1–0.2 ml) were spotted on 3MM Whatman filter paper (Whatman, Maidstonem, UK), air dried, individually kept in zip-lock plastic bags with desiccants and were sent to the Parasitology Unit Laboratory for DNA extraction from the first investigation. For the second investigation, approximately 1–5 ml of venous blood was drawn in a syringe and transferred to a vacuum tube containing anticoagulant (EDTA). EDTA blood samples were stored in a 4 °C refrigerator until being processed. The remaining blood in the syringe was spotted on 3MM Whatman filter papers, air dried and individually kept in zip-lock plastic bags with desiccants for future use. For each of the finger-prick and venous blood sample, a portion of the blood was utilized directly to prepare thick and thin blood smears for microscopic analysis in both investigations. All individuals and all age groups were invited to participate in the study. Signed informed consents were obtained from all participants before enrolment. Interviews were performed with parents and/or legal guardians for participants 7 years or younger. The participant’s history of previous malaria infection, occupation, age and gender, were recorded at the time of enrolment for the second set of sampling.

### Microscopic examination

Thin and thick blood films for the initial investigation were prepared and examined by personnel and microscopists from the Sarawak Health Department, MOH. On the other hand, thin and thick blood films from the second investigation were prepared in separate slides and labelled accordingly at the sites. Thin blood films were dried and fixed using absolute methanol while thick blood films were left to dry thoroughly. Both blood films were stained with 3% Giemsa for 45 min to 1 h. Blood films were examined by two trained microscopists who had over 10 years of experience in reading malaria slides and their proficiency were periodically evaluated by local/regional quality assurance programs of the Ministry of Health Malaysia. Identification of malaria species was done using the thin blood smear while parasitaemia was examined using thick blood smear.

### DNA extraction

DNA was extracted from DBS collected in the initial study using the QIAGEN DNeasy^®^ Blood & Tissue Kit following manufacturer’s protocol with slight modification. Three 3-mm^2^ filter paper punches, equivalent to approximately 10–15 µl blood was excised using a puncher and transferred into a 1.5 μl tube individually. DNAs from 200 µl of venous blood samples collected in Vacutainer^®^ EDTA tubes on the second study were extracted using the QIAamp^®^ Blood Kit following manufacturer’s recommendation.

### Detection of malaria species by PCR

The nested PCR assay was performed on samples from the pilot study as previously published by Snounou et al. [6] and Singh et al. [8, 12] with slight modification. Five microliter of DNA was used as template in the first amplification process (nest 1) and subsequently 2 µl if the nest 1 product was used as template in nest 2 amplification. The method is based in the amplification of a region of the small subunit of the ribosomal gene (18S RNA) of *Plasmodium* species and the primers are listed in Table 1. Known positive and negative samples from previous malaria diagnosed or uninfected individuals were used as controls. The semi-nested multiplex PCR (NM-PCR) was performed on samples from the second study as previously described [13–15] with slight modification. Five microliter of DNA was used as template in the first amplification process. The method is based in the amplification of a region of the small subunit of the ribosomal gene (18S rRNA) of form A (asexual) of *Plasmodium* species. This method is a semi-nested multiplex PCR using a single reaction for the second amplification with a mixture of five specific primers for each human and one zoonotic *Plasmodium* species, and a universal *Plasmodium* primer. The nucleotide sequence (Table 2) of the primer PLF and NewPLFshort is identical for all *Plasmodium* species including the ones which infect human, monkeys, other mammalian, birds and other vertebrates. Uninfected human blood was used as negative control.

**Table 1:** List of primers including name, nucleotide sequence, PCR step, fragment size and specificity of the nested PCR [6, 8, 12]

	*Plasmodium* spp.	Size (bp)	Primer name	Primer sequences	PCR (T)
Nested 1st round	Genus-specific	1600–1700	rPLU1 rPLU5	TCAAAGATTAAGCCATGCAAGTGA CCTGTTGTTGCCTTAAACTTC	56 °C
Nested 2nd round	Genus-specific	235	rPLU3 rPLU4	TTTTTATAAGGATAACTACGGAAAAGCTGT CCCGTCATAGCCATGTTAGGCCAATACC	59 °C
Nested 2nd round species specific	*P. vivax*	121	rVIV1 rVIV2	CGCTTCTAGCTTAATCCACATAACTGATAC ACTTCCAAGCCGAAGCAAAGAAAGTCCTTA	60 °C
	*P. falciparum*	206	rFAL1 rFAL2	TTAAACTGGTTTGGGAAAACCAAATATATT ACACAATGAACTCAATCATGACTACCCGTC	
	*P. ovale*	226	rOVA1 rPLU2	ATCTCTTTTGCTATTTTTTAGTATTGGAGA ATCTAAGAATTTCACCTCTGACATCTG	
	*P. malariae*	145	rMAL1 rMAL2	ATAACATAGTTGTACGTTAAGAATAACCCC AAAATTCCCATGCATAAAAATTATACAAA	
	*P. knowlesi*	153	Pmk8 Pmkr9	GTTAGCGAGAGCCACAAAAAAGCGAAT ACTCAAAGTAACAAAATCTTCCGTA	61 °C

**Table 2:** List of primers including name, nucleotide sequence, final concentration, PCR step, fragment size and specificity of the NM-PCR [13–15]

Name	Sequence 5′-3′	Final concentration (μM)	PCR (T^a^)	Size^a^ (bp)	Specificity
PLF	AGTGTGTATCAATCGAGTTTC	0.0375	1° PCR 58 °C	783–821^b^	*Plasmodium*
REV	GACGGTATCTGATCGTCTTC	0.0375	1° PCR 58 °C	–	Universal
HUF	GAGCCGCCTGGATACCGC	0.00625	1° PCR 58 °C	231	Human
NewPLFshort	CTATCAGCTTTTGATGTTAG	0.0375	2° PCR 53 °C	–	*Plasmodium*
Malshort	TCCAATTGCCTTCTG	0.0625	2° PCR 53 °C	215	*P.* *malariae*
Falshort	GTTCCCCTAGAATAGTTACA	0.0375	2° PCR 53 °C	344	*P.* *falciparum*
Vivshort	AAGGACTTCCAAGCC	0.025	2° PCR 53 °C	457	*P.* *vivax*
OvaNew	CCAATTACAAAACCATG	0.0875	2° PCR 53 °C	176	*P.* *ovale*
NewPKrev	CGCGGAGGCATC	0.025	2° PCR 53 °C	389	*P.* *knowlesi*

^a^Expected size of the PCR product between the corresponding Universal/*Plasmodium* primer with the species specific primer

^b^Size depending upon species

## Results

### Sampling characteristics

A total of 1744 DBS were received from the Sarawak Health Department in 2013, together with microscopic examination results, previously read by microscopists from the Sarawak Health Department. Data on age, gender, exposure and travel history or occupation were considered confidential thus not provided to us (Parasitology Unit, IMR). The DBS were collected from 8 localities in the district of Belaga (Table 3). A total of 251 whole blood in EDTA and Giemsa-stained blood films from two localities (Long Malim and Long Wat) in the District of Belaga, Sarawak in 2014 were brought back to the Parasitology Unit of the Institute for Medical Research Kuala Lumpur for laboratory diagnosis. The study population consisted of infants to pre-schooled children (1%), school children (12%) and those above 17 years old (87%) who are either household income earners or housewives (Table 4). The male to female ration this study was 1.1 male to 1 female. Most female above the age of 17 (86%) are housewives while male above the age of 17 and below the age of 70 (82%) are mostly farmers and loggers, while some works at the oil palm plantation. Children within the age 7 to 16 are mostly in school located near to their longhouses. None of the tribal villagers from both studies reported malaria-like illness and appeared reasonably healthy during blood sampling activity.

**Table 3:** Prevalence of submicroscopic malaria from bloodspot received from Belaga District in 2013 confirmed by nested PCR

Locality in Belaga district	No. of bloodspots received	Malaria positive by microscopy	Nested PCR assay		Proportion PCR (%)
			*P. vivax*	Other *Plasmodium* sp.	
Long Malim	383	0	9	0	0.5
Long Wat	90	0	3	0	0.2
Long Jaik	173	0	3	0	0.2
Long Tanyit	177	0	1	0	0.1
Lusong Laku/SK Lusong Laku	567	0	4	0	0.2
Rh Udau Tedong	126	0	5	0	0.3
Uma Pawa Tr Eric	77	0	0	0	0
Simpang Uma Nyaving	151	0	2	0	0.1
Belaga distrct	1744	0	27	0	1.5

**Table 4:** Prevalence of submicroscopic malaria from study samples collected from Long Malim and Long Wat in 2014 confirmed by semi-nested multiplex PCR

Variable Factor	Long Malim			Long Wat			Total		
	No. of sample tested	PCR positive	PCR positive (%)	No. of sample Tested	PCR positive	PCR positive (%)	No of sample Tested	pcr positive	PCR positive (%)
Gender									
Female	53	31	58.5	62	35	56.5	115	66	57.4
Male	59	24	40.7	77	28	36.4	136	52	38.2
Age group									
0–5 y/o (pre-school children)	0	0	0	1	0	0	1	0	0
6–16 y/o (School children)	12	7	58.3	18	10	55.6	30	17	56.7
17 ≥, (household income earners/housewives)	100	48	48.0	120	53	44.2	220	101	45.9
PCR-confirmed malaria species									
*P. falciparum*	55	26	47.2	63	24	38.1	118	50	42.4
*P. vivax*		18	32.7		21	33.3		39	33.1
*P. knowlesi*		9	16.4		0	0		9	7.6
Mixed *Pf *+ *Pv*		1	1.8		16	25.4		17	14.4
Mixed *Pv *+ *Pk*		1	1.8		2	3.2		3	2.5
Total	112	55	49.1	139	63	45.3	251	118	47.0

### Confirmation of submicrosopic malaria by nested PCR assays

No malaria parasites were seen by microscopic examination of Giemsa-stained thick and thin blood films collected from the 2013 and 2014 studies (Tables 3 and 4). Nested PCR protocol detected 27 (1.6%) *P. vivax* and none were positive for other malaria species. Sources of DBS samples received were from 8 localities listed in Table 4. Long Malim had the highest prevalence of *Plasmodium* infections; 0.5% (9/1744), followed by Rh. Udau Tedong, Lusong Laku/SK Lusong Laku, Long Wat, Long Jaik, Simpang Uma Nyaving and Long Tanyit with 0.3% (5/1744), 0.2% (4/1744). 0.2% (3/1744), 0.2% (3/1744), 0.1% (2/1744) and 0.05% (1/1744), respectively. From the total of 251 blood samples obtained in the second study, 118 (47%) samples were tested positive for *Plasmodium* sp. by PCR (Table 4). Interestingly, malaria infection attributed to not only *P. vivax*, but also to *P. falciparum* and *P. knowlesi* were found. Mono-infections of *P. vivax*, *P. falciparum* or *P. knowlesi* were in proportions of 15%, 20% and 4%, respectively; while mixed infection of *P. vivax* and *P. falciparum* accounted for 7% and mixed infection of *P. vivax* and *P. knowlesi* accounted for 1% of total blood samples tested by PCR. In Long Malim, the most prevalent species was *P. falciparum* with 26 cases (47.2%), followed by *P. vivax*, *P. knowlesi* and mixed infections with 18 (32.7%), 9 (16.4%) and 2 (3.6%), respectively. In Long Wat, the prevalence of *P. falciparum* was also the highest with 24 cases (38.1%) followed by *P. vivax* and mixed infections with 21 (33.3%) and 18 (28.6%), respectively. There were no mono-infections of *P. knowlesi* detected from Long Wat. There is no significant difference between the prevalence of malaria parasites in both localities (P = 0.55). Within Long Malim (P = 0.22) and Long Wat (P = 0.32), there was no significant difference between the prevalence of malaria species in both gender. Both genders are equally at risk of harbouring submicroscopic malaria within the population.

## Discussion

Currently, the Malaysia is in the elimination phase of the human-only *Plasmodium* species and proposes to eliminate malaria by the year 2020 based on World Health Organization (WHO) efforts, and the Malaysian National Malaria Control Programme. In 2018, Malaysia had approximately 4630 malaria cases, where *P. knowlesi* accounted for 89% of the total number of cases reported and 11% for the human malaria cases (imported and introduced). The substantial decrease of *P. falciparum* and *P.* vivax cases gives rise to *P. knowlesi*, and this trend threatens malaria elimination. Although SMM have been reported from Southeast Asian countries (Thailand, Indonesia, Myanmar, Cambodia and Vietnam) with the prevalence rate of between 1.7 and 36% [5, 16–22], limited data about such carriage are reported in Malaysia. Only two studies in Malaysia has reported prevalence of asymptomatic SMM [5, 18]. In addition to that, with the presence *P. knowlesi*, a zoonotic malaria parasite maintained by macaques, that has been described throughout Southeast Asia is now the most common cause of human malaria in Malaysian Borneo [23]. In 2013 and 2014, the number of malaria cases reported in Kapit Division is 204 and 219 respectively. In 2013, there were 10 cases of *P. falciparum*, 170 cases of *P. knowlesi*, 4 cases of *P. malariae* and 7 cases of *P. vivax.* The number increased substantially in 2018 with 51 cases of *P. falciparum*, 267 *P. knowlesi*, and 43 cases of *P. vivax.* This data shows that despite efforts taken to control and prevent the disease, the number is still increasing. However, there is lack of evidence for or against a substantive role of submicroscopic malaria for transmission in this country. This makes the ethics of treating asymptomatic and submicroscopic parasitaemia unclear and dependent on the risk–benefit ratios of the treatment and/or prevention strategies being implemented. While Malaysia has a malaria elimination goal of zero indigenous transmission for all human malaria species by 2020, achieving malaria elimination requires targeting the human reservoir of infection, including those with asymptomatic infection which requires the combination of universal coverage of interventions with implementation of a robust surveillance system that collects, transmits, and analyses data about cases and programme activities in real time to inform rapid response strategies. The current study adds to the available evidence on the presence of submicroscopic parasitaemia in this country. Accurate diagnosis of asymptomatic SMM is important for providing realistic estimates of malaria burden and preventing misinformed interventions. However, it is not an easy task as low levels of parasitaemia may act as silent reservoir of transmission thus remains infectious to susceptible mosquito vectors. In this study, Since the transmission of malaria parasites from humans to mosquitoes requires the presence of gametocytes, any strategy that interferes with the development or persistence of gametocytes should help to interrupt transmission [4, 24–26]. In most malaria endemic areas, the majority of parasite carriers are asymptomatic. Asymptomatic individuals carrying gametocytes remain available as a reservoir for transmission by mosquitoes, contributing to the persistence of malaria transmission within local populations. It is important to carry out more research to understand better the contribution of submicroscopic infections in malaria transmission in low endemic settings and to identify which diagnostic strategies are most reliable. This can be done, either by retrospective molecular analysis of prior cohort studies where only microscopy was used, or prospectively, in the context of cohort studies and national surveys, in which microscopy-negative but PCR-positive subjects are followed and not treated unless they become symptomatic or develop patent parasitaemia [27].

While a systematic review indicated a relationship between microscopy and polymerase chain reaction (PCR) prevalence, the proportion of all infections in a population which are submicroscopic is not predictable in any given situation, particularly in areas of low transmission. Thus, when quantification of submicroscopic infections is needed, this has to be measured directly. Submicroscopic cases are often associated with asymptomatic malaria parasite carriers especially adults. Microscopic diagnosis using thick and thin blood smears plays an important role in malaria diagnosis because of its ability to diagnose and differentiate each species of malaria, and so it is used as the gold standard for any new detection tool or technique [28]. Nonetheless, microscopy has been found to have a great number of limitations. A superior and a more reliable diagnostic technique need to be put in place to enable proper treatment and control of malaria. A good microscopist can identify 50 parasites per µl blood (p/µl) while the expert microscopist will struggle to detect regularly infections < 20 p/µl (Chiodini, pers. commun.). Furthermore, the diagnostic accuracy relies on the quality of the blood smear and experience of laboratory personnel. Malaria microscopy is time consuming, requires skilled laboratory technicians and is often subject to unreliable results from different laboratories [29]. In recent years, alternative methods to identify malaria infections with varying degrees of specificity and sensitivity have been developed. These include fluorescence microscopy of malaria parasites stained with acridine orange, dipstick immunoassays that detect species-specific parasite antigens, and, more recently, detection of parasite nucleic acids after amplification by PCR. With some of these methods, sensitivities and specificities approaching and even exceeding those of the thin and thick film can be attained [30].

The WHO recommends prompt parasitological confirmation of diagnosis by either good quality microscopy or good quality RDTs before treatment is being administered [31]. Nonetheless, many of these asymptomatic infections are present at densities below the limit for microscopic detection and, therefore, use of microscopy or RDT is likely to lead to underestimation of the malaria burden [32, 33]. In developing world where malaria is highly prevalent, the resources to aid in proper and accurate diagnosis are lacking and has led to improper administration of anti-malarial drugs [34]. A number of different PCR diagnostic techniques exist: single step, nested, multiplex and quantitative. Alternative nucleic acid amplification (NAA) techniques have been developed which do not need thermocyclers, the most common are loop-mediated isothermal amplification (LAMP) and nucleic acid sequence-based amplification (NASBA). In this study, the nested PCR [6, 8, 12] and semi-nested multiplex PCR [13–15] assays were used with slight modification. The nested PCR assay has a limit of detection of at least 6 parasites/µl for blood spots. It has a much higher sensitivity compared to the single step PCR for four major *Plasmodium* species. Meanwhile, the semi-nested multiplex method developed by Rubio et al. [13–15] has a limit of detection of 0.01 parasites/µl of blood and is able to detect all human and knowlesi malaria simultaneously. The villagers of Long Malim and Long Wat in the initial study were relocated to a new resettlement (Tegulang Resettlement Site) approximately 20 km from their original locality within the District of Belaga under the Murum Resettlement Scheme. In this study, there is a large discrepancy in prevalence from the study samples in 2013 to the 2014 cohort tested (1.65 vs. 47%). This could not merely be due to the better limit of detection (LOD) seen in the semi-nested multiplex PCR as compared to the method used in the initial study (6 parasites/µl of blood), but the major contributing reason would appear to be the blood volumes for which DNA was extracted from i.e. filter paper (10–15 µl) as opposed to whole blood (200 µl). Further studies should be carried out in these areas as there are various factors to the difference of incidence of asymptomatic SMM in both studies. This could possibly indicate that there is high prevalence of submicrosopic and asymptomatic malaria infections in Belaga or due the fact that two different method of detection (nested PCR vs semi-nested multiplex PCR) and two different samples (dried blood spots vs whole blood) were collected during the first and second study, respectively. In 2013, 2 of the villages (Long Malim and Long Wat) where the initial sampling took place were displaced to give way to the Murum Hydro-Electric Power Project. The communities of Long Wat, Long Malim (Penan) and the Long Malim (Kenyah) have agreed to be resettled at the Tegulang River, which was where the second sampling took place. The villagers of Long Malim and Long Wat comprises of the Penan and Kenyah ethnic communities. The economy of Murum Penan communities consists of farming and forest based activities such as hunting, fishing and gathering of forest products while the Kenyah community at Long Malim have long been living a settled life centred on agriculture activities such as planting hill paddy [35].

In general, challenges in developing multiplex PCR systems include difficulties in designing primers and optimal conditions for a highly sensitive and specific assay. The specificity of the primers for each species is essential in order to get good results in the identification of each species and avoid false negatives and misidentifications [14, 36–38]. In a malaria epidemiological survey, especially in low transmission area such as Belaga, a substantial proportion of malaria infections are missed by microscopy and/or RDTs because of low parasite-density infections. Generally, the use of highly sensitive diagnostic tools should be considered only in low-transmission settings where there is already widespread malaria diagnostic testing and treatment and low parasite prevalence rates (e.g. < 10%). It is also important to note that submicroscopic *P. falciparum* and *P. vivax* infections are common in both low and high transmission settings. Use of NAA methods in malaria programmes should be considered for epidemiological research and surveys to map sub-microscopic infections in low transmission areas. NAA methods might also be used for identifying foci for special interventions in elimination settings. According to Okell et al. [32], there is an association between transmission intensity and the proportion of infections that are submicroscopic. Their findings suggests that, as the underlying PCR prevalence increased, the microscopy: PCR prevalence ratio also increased (the prevalence ratio was 1.135 times higher per 10% increase in PCR prevalence [95% CI 1.051–1.226]; P = 0.002). This finding also suggests that a higher proportion of infections are undetected by microscopy in areas with low levels of transmission. Thus, in studies where the PCR prevalence in the study population was < 10%, microscopy detected only 12.0% of the infections that were identified by PCR (95% CI 4.8–29.9%), whereas in studies in which the PCR prevalence was ≥ 75%, microscopy detected 74.5% of infections (95% CI 67.1–82.8%). However, submicroscopic infections are of particular relevance in low-transmission areas aiming for elimination, where they are likely to sustain transmission if not detected [32] and the main problem with PCR is contamination with rates of 0.7–10% reported by laboratories [39], and it would be expected to have the biggest effect in low-transmission settings, where a higher proportion of samples have true-negative results. There is a need for development of a standardized PCR for parasite detection especially in low transmission areas. Another experimental explanation for the observed association could be that microscopists are less experienced in identifying malaria in settings where transmission is lower.

Asymptomatic SMM has rarely been a major research focus. In malaria endemic areas, asymptomatic malaria parasite carriers especially adults are not uncommon and, as potential gametocyte carriers, represent an important reservoir for malaria transmission [40]. Asymptomatic SMM may serve as a reservoir for infection even when very efficient rapid diagnosis and treatment programmes have been implemented [41]. In malaria endemic areas, continuous exposure to *Plasmodium* parasites leads to asymptomatic carriers that provide a fundamental reservoir of parasites, contributing to the persistence of malaria transmission [42]. Particularly, subpatent malaria is still transmissible and will complicate elimination of malaria in high transmission regions. A major obstacle in the study of asymptomatic malaria is the lack of standard diagnostic criteria. Quantifying parasitaemia, rather than documenting presence or absence of parasites, may also be an important consideration when diagnosing asymptomatic malaria. However, a universally standard parasite threshold for classifying an infection as asymptomatic has yet to be defined, as different studies use variable cut-off levels for parasite density [23, 43, 44]. Though the use of species-specific PCR possible is not always available in the field, or even perhaps practical for testing infections that are negative by microscopy, it is a powerful tool for finding asymptomatic malaria within a population. Various studies found that as many as two-thirds of the microscopy-negative patients had subpatent levels of parasites determined by diagnostic PCR, indicating that almost the entire population was chronically infected with asymptomatic malaria [45–48]. There is some indication that very low-density *P. vivax* infections do contribute to ongoing transmission but data are very limited [49, 50]. It is also apparent that RDT and microscopy do not detect the full prevalence of *P. vivax* infection because of the large proportion of submicroscopic infection in low transmission settings.

Due to time constraint, the detection of gametocytaemia of *P. falciparum* and *P. vivax* were not carried out. Since the transmission of malaria parasites from humans to mosquitoes requires the presence of gametocytes, any strategy that interferes with the development or persistence of gametocytes should help to interrupt transmission. In most malaria endemic areas, the majority of parasite carriers are asymptomatic. Asymptomatic individuals carrying gametocytes remain available as a reservoir for transmission by mosquitoes, contributing to the persistence of malaria transmission within local populations. It is vital to determine the gametocyte carriage potential of individuals followed without treatment and to identify surrogate markers of latent *P. vivax i*nfection because currently there is no diagnostic tool which is able to directly detect latent *P. vivax*. Studies of duration of infection, submicroscopic gametocyte carriage, and gametocyte production potential need to be carried out in submicroscopic infections in low transmission settings where elimination is being considered. This can be done, either by retrospective molecular analysis of prior cohort studies where only microscopy was used, or prospectively, in the context of cohort studies and national surveys, in which microscopy-negative but PCR-positive subjects are followed and not treated unless they become symptomatic or develop patent parasitaemia.

For malaria elimination settings it is critical to detect all infections, including those with low and sub-microscopic parasite densities in asymptomatic carriers as they represent a parasite reservoir in the community capable of effectively transmitting infections to mosquitoes [4, 25] and seeding transmission foci [10]. It is well known that malaria epidemiology varies between country and region, and particularly between islands, because the dominant vector species, the characteristics of human populations and factors that influence transmission such as rainfall, temperature, housing conditions and population movement differ. Therefore, the challenges to malaria elimination in different settings will vary. Each area needs to investigate the malaria epidemiology and carefully tailor its diagnosis strategy to the local context. In part, the use of molecular assays such as PCR to detect parasite DNA has improved the sensitivity of diagnostics to find subpatent (i.e. below the detection limits for microscopy) infections that are more likely to be asymptomatic, and this has contributed to the understanding of the extent of the asymptomatic parasite reservoir [33]. As the magnitude of the asymptomatic parasite reservoir has been revealed through increasing use of more sensitive molecular diagnostic methods, new strategies to target individuals with silent infections are being developed and evaluated.

## Conclusion

Submicroscopic infections are thought to be important contributors to maintaining malaria transmission. However, there is currently no direct evidence that specifically targeting this low-density parasite reservoir will hasten progress towards elimination. In most malaria endemic settings, asymptomatic infections outnumber symptomatic infections. The objective of this study was to detect the presence of submicroscopic malaria in low transmission area. It was achieved with by using molecular methods. The semi-nested multiplex PCR has proven to be a very useful tool in detection of asymptomatic submicroscopic malaria in both high and low transmission area. However, the WHO does not currently provide guidance to countries regarding the programmatic suitability of molecular diagnostics or guidelines on how to utilize the information that would emerge from their use. Quality assured microscopy is still officially considered the gold standard by the WHO, despite large bodies of evidence that shows that PCR and other nucleic acid amplification-based assays are more sensitive than microscopy. There is a need to develop guidance on indications for use, assay selection and quality assurance/control for PCR and other molecular diagnostic techniques for the specific conditions in which use of these malaria diagnostic tools may be appropriate. In this study, *P. knowlesi* infections were also detected among the population. It remains unknown if and how much humans contribute to the infectious reservoir for *P. knowlesi* transmission. While the submicroscopic parasite densities of other malaria species have been shown to be capable of infecting mosquitoes, all experimental infections with *P. knowlesi* have been from clinical malaria cases. As demonstrated by the data from this study, the most common cause of asymptomatic SMM is *P. falciparum* and followed *P. vivax*, despite the fact that *P. knowlesi* is present. These results illustrate the possibility that by the time a person is symptomatic, the parasite load would have risen by many folds. This is an important aspect to be considered by public health personnel as well as clinicians on whether there is need to heighten surveillance or to make PCR detection routine. The occurrence of other possible zoonotic species also needs to be further elucidated. Unfortunately, this study did not cover that aspect and since this is one of the first community-based studies, more studies needs to be conducted to confirm this. Entomological and primatological studies are also needed to evaluate the presence of potential *P. knowlesi* vectors in these village environments and the proximity of infected reservoirs.

## Abbreviations

PCR: polymerase chain reaction
SMM: submicroscopic malaria
ITN: insecticide-treated nets
WHO: World Health Organization
EDTA: ethylenediaminetetraacetic acid
DNA: deoxyribonucleic acid
RNA: ribonucleic acid
DBS: dried blood spots
