# Correction to: Evidence of asymptomatic submicroscopic malaria in low transmission areas in Belaga district, Kapit division, Sarawak, Malaysia

**DOI:** 10.1186/s12936-019-3005-6

**Published:** 2019-11-21

**Authors:** Adela Ida Jiram, Choo Huck Ooi, José Miguel Rubio, Shamilah Hisam, Govindarajoo Karnan, Nurnadiah Mohd Sukor, Mohd Mafe Artic, Nor Parina Ismail, Nor Wahida Alias, Ismail Aziah

**Affiliations:** 1 Parasitology Unit, Infectious Diseases Research Centre, Institute for Medical Research, Ministry of Health Malaysia, Jalan Pahang, 50588 Kuala Lumpur, Malaysia; 2 Institute for Research in Molecular Medicine (INFORMM), Universiti Sains Malaysia, 16150 Kubang Kerian, Kelantan Malaysia; 3 Vector Borne Diseases Section, Sarawak Health Department, Ministry of Health Malaysia, Diplomatik Road, Off Bako Road, Petra Jaya, 93050 Kuching, Sarawak Malaysia; 4 Malaria & Emerging Parasitic Diseases Laboratory, Parasitology Department, National Centre of Microbiology, Instituto de Salud Carlos III (ISCIII), Carretera de Majadahonda - Pozuelo, km. 2,200, Majadahonda, 28220 Madrid, Spain

## Correction to: Malar J (2019) 18:156 10.1186/s12936-019-2786-y

Please note that an author has been erroneously omitted from the author list of the published article [1].

The missing author (Ismail Aziah) was the supervisor of the corresponding author, and contributed in terms of review and discussion of the content of the manuscript.

Furthermore, please note that one of the affiliations of the corresponding author has been missed: the corresponding author is affiliated to affiliation ‘2’, in addition to affiliation ‘1’.

Please find the corrected author list in this article.

The authors apologize for any inconvenience caused.
